# Preservation of General Intelligence following Traumatic Brain Injury: Contributions of the Met66 Brain-Derived Neurotrophic Factor

**DOI:** 10.1371/journal.pone.0088733

**Published:** 2014-02-26

**Authors:** Aron K. Barbey, Roberto Colom, Erick Paul, Chad Forbes, Frank Krueger, David Goldman, Jordan Grafman

**Affiliations:** 1 Decision Neuroscience Laboratory, University of Illinois, Urbana, Illinois, United States of America; 2 Beckman Institute for Advanced Science and Technology, University of Illinois, Urbana, Illinois, United States of America; 3 Department of Internal Medicine, University of Illinois, Champaign, Illinois, United States of America; 4 Department of Psychology, University of Illinois, Champaign, Illinois, United States of America; 5 Department of Speech and Hearing Science, University of Illinois, Champaign, Illinois, United States of America; 6 Neuroscience Program, University of Illinois, Champaign, Illinois, United States of America; 7 Universidad Autónoma de Madrid, Fundación CIEN/Fundación Reina Sofía, Madrid, Spain; 8 Department of Psychology, University of Delaware, Delaware, Maryland, United States of America; 9 Department of Molecular Neuroscience, George Mason University, Virginia, United States of America; 10 Laboratory of Neurogenetics, National Institute on Alcohol Abuse and Alcoholism, National Institutes of Health, Bethesda, Maryland, United States of America; 11 Traumatic Brain Injury Research Laboratory, Rehabilitation Institute of Chicago, Chicago, Illinois, United States of America; Rutgers University, United States of America

## Abstract

Brain-derived neurotrophic factor (BDNF) promotes survival and synaptic plasticity in the human brain. The Val66Met polymorphism of the BDNF gene interferes with intracellular trafficking, packaging, and regulated secretion of this neurotrophin. The human prefrontal cortex (PFC) shows lifelong neuroplastic adaption implicating the Val66Met BDNF polymorphism in the recovery of higher-order executive functions after traumatic brain injury (TBI). In this study, we examined the effect of this BDNF polymorphism on the preservation of general intelligence following TBI. We genotyped a sample of male Vietnam combat veterans (*n* = 156) consisting of a frontal lobe lesion group with focal penetrating head injuries for the Val66Met BDNF polymorphism. Val/Met did not differ from Val/Val genotypes in general cognitive ability before TBI. However, we found substantial average differences between these groups in general intelligence (≈ half a standard deviation or 8 IQ points), verbal comprehension (6 IQ points), perceptual organization (6 IQ points), working memory (8 IQ points), and processing speed (8 IQ points) after TBI. These results support the conclusion that Val/Met genotypes preserve general cognitive functioning, whereas Val/Val genotypes are largely susceptible to TBI.

## Introduction

Traumatic Brain Injury (TBI) is a global public health epidemic. In the US alone, more than 3 million people sustain a TBI annually. It is one of the most disabling injuries as it results in motor and sensory deficits as well as severe cognitive, emotional, and psychosocial impairment. Fueled by the recognition of TBI as the “signature injury” in our wounded soldiers in Iraq and Afghanistan and its often devastating impact on athletes playing contact sports, interest in TBI has increased exponentially. Unfortunately, despite increased awareness of its detrimental consequences, there has been little progress in developing effective TBI interventions. Following TBI, the brain attempts to activate repair mechanisms and stimulate neuroregeneration, which may be facilitated by the presence of a unique family of neurotrophic factors, including nerve growth factor, glia-derived neurotrophic factor, neurotrophin-3, and brain-derived neurotrophic factor (BDNF). In this study, we build upon our prior work [1], [2] by investigating the relationship between variations in the BDNF gene and preservation of general intelligence in the prefrontal cortex (PFC) after TBI. Damage to the PFC leads to impairment in executive function, which normally allows individuals to effectively engage in complex goal-directed behaviors, whereas the domains of perception and language are more often preserved [3].

BDNF has emerged as a major regulator of synaptic connections [4], synaptic plasticity [5], [6], and neural survival and growth [7], [8]. Within the BDNF gene, a distinct haplotype containing a frequent single-nucleotide polymorphism (SNP), located at nucleotide 196 (dbSNP rs6265), produces a G-to-A substitution, which results in a valine-to-methionine (Val66Met) substitution at codon 66 in the propeptide of the BDNF molecule [9], [10]. This SNP alters the intracellular tracking and packaging of pro-BDNF, affecting the regulated secretion and neuroplastic effect of mature BDNF [11].

The Val66Met BDNF polymorphism has been linked to cognitive functioning and clinical pathology [12]. In healthy populations, the methionine (Met) allele has been linked to impaired episodic memory, working memory, and hippocampal function [11], [13]–[16], although a recent study showed a functional advantage for the Met allele when cognitive control, such as response inhibition, is required [17]. Furthermore, the Met allele has been associated with lower hippocampal levels of N-acetylaspartate [11] and less gray matter volume throughout the PFC and middle temporal lobes as well as limbic structures such as the amygdala [16], [18]–[20]. In clinical populations, the Met allele has been associated with a wide range of neurodegenerative and psychiatric disorders such as Alzheimer’s disease [21]–[23] and bipolar disorder [24], [25], arguing that a common clinical symptom of these disorders is a varying degree of impairment in higher cognitive abilities [26], [27].

To our knowledge, the way in which the Val66Met BDNF polymorphism affects the preservation of general intelligence after TBI has not been systematically examined. Recent evidence suggests that this approach can be productively applied to understand recovery of executive function [1] and cognitive performance following TBI [28], and motivates the present more comprehensive investigation of general intelligence. A central aim of the current effort is to investigate the way in which the Val66Met BDNF polymorphism affects the preservation of specific facets of human intelligence, advancing prior research by administering a comprehensive assessment of general intelligence and applying latent variable modeling to examine key facets of intellectual ability (i.e., verbal comprehension, perceptual organization, working memory, and processing speed). Here, we genotyped a sample of male Vietnam combat veterans with focal penetrating TBI and administrated the Wechsler Adult Intelligence Scale to examine specific competencies for general intelligence. Importantly, veterans with different genotypes did not show any difference in general intelligence before TBI.

## Materials and Methods

### Participant Data

Participants were drawn from the Phase 3 Vietnam Head Injury Study (VHIS) registry, which includes American male veterans who all suffered brain damage from penetrating head injuries in the Vietnam War (*n* = 171). This study was approved by the National Naval Medical Center Institutional Review Board and, in accordance with stated guidelines, all subjects read and signed informed consent documents. Since our participants have had an injury that may have impaired their ability to think clearly and make decisions, we ask that they travel with a primary caregiver and name them as a Durable Power of Attorney for research and medical care at NNMC. Phase 3 testing occurred between April 2003 and November 2006.

### Genotyping

156 participants were genotyped for the single-nucleotide (G196A) polymorphism (SNP) of the BDNF gene that is located on chromosome 11p13 [33]. The Val66Met polymorphism of the BDNF gene (dbSNP identifier: rs6265; GenBank accession number 2174122) is a G-to-A substitution, which results in replacement of the Val at codon 66 of the BDNF protein by Met. Individuals who are G/G homozygous produce only the Val-containing isoform of the proBDNF protein, A/A homozygous individuals produce only the Met-containing isoform of proBDNF, and G/A heterozygous individuals produce both protein isoforms. Note that groups with Met/Val and Met/Met genotypes were combined for statistical analyses into a Met/− BDNF group (frontal lobe lesion group: Val/Val = 97, Val/Met = 56, Met/Met = 3), since the frequency of the Met/Met genotype was low and the Met/Met-containing BDNF molecules are functionally equivalent to Met/Val BDNF proteins [9].

Genomic DNA was isolated from blood leukocytes using a Nucleon BACC2 kit according to the manufacturer’s protocol (GE Healthcare Life Science). Quality and quantity of genomic DNA were determined spectrophotometrically using the absorbance reading at 260 and 280 nm. Some DNA samples were repurified by incorporating an additional phenol/chloroform (24∶1 v/v) extraction before recovery by ethanol precipitation. DNA concentrations were measured using a NanoDrop ND-1000 spectrophotometer (NanoDrop Technologies). The completion rate of each assay was >99%, with an error rate of <1%. Val66Met BDNF genotypes at rs6265 were determined using a 5′-exonuclease allelic discrimination (TaqMan) assay using Reference SNP ID: rs6265 (ABI Assay on Demand C_11592758_10; Applied Biosystems), on an ABI7900 instrument. Genotyping error rate for this assay was determined by replicate genotyping of samples and was <0.005.

Participants were also genotyped for the catechol-O-methyltransferase (COMT) Val158Met polymorphism (GenBank accession no. Z26491) that has also been associated with the modulation of executive functioning [34], [35]. A 5′ nuclease assay using fluorogenic detection probes was performed based on the G1947A single nucleotide polymorphism within exon 4 of theCOMTgene (NCBI nucleotide accession number Z26491), corresponding to codon 158 of the COMT gene (NCBI accession number BC011935). The detection oligonucleotide sequences were as follows: 5′-Fam6-CCTTGTCCTTCAcGCCAGCGA-TAMRA-3′ (Val158 detection probe) and 5′-Vic-ACCTTGTCCTTCAtGCCAGCGAAAT-TAMRA-3′ (Met158 detection probe). FAM is 6-carboxyfluorescein, and TAMRA is 6-carboxytetramethylrhodamine. The variant nucleotide in each detection probe is shown in lowercase. The oligonucleotide primers used for amplification were 5′-TCGAGATCAACCCCGACTGT-3′ (forward) and 5′-AACGGGTCAGGCATGCA-3′ (reverse). Target DNA amplification, fluorescence measurements, and allele discrimination were accomplished using a ABI 7900 Sequence Detection System (Applied Biosystems).

### Lesion Analysis

**Figure 1:**
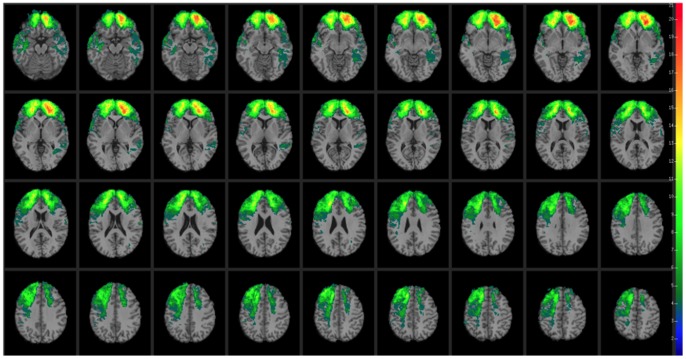
Lesion mapping results for Val/Val genotype patients (*n* = 97). In each axial slice, the right hemisphere is on the reader’s left.

**Figure 2:**
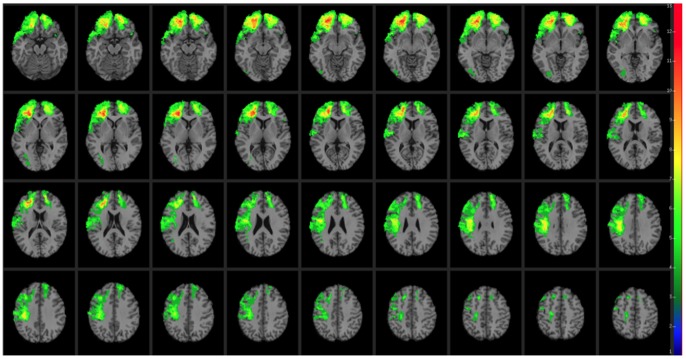
Lesion mapping results for Val/Met genotype patients (*n* = 59). In each axial slice, the right hemisphere is on the reader’s left.

**Figure 3:**
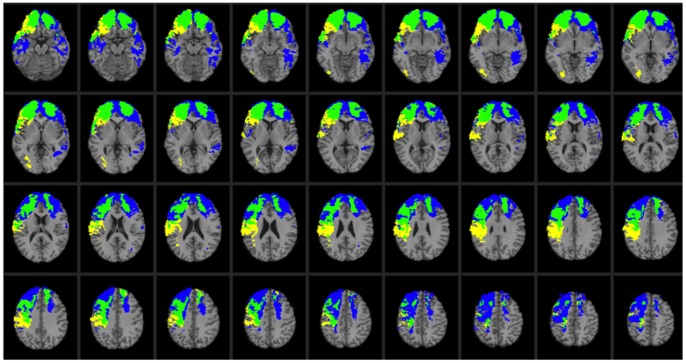
Lesion overlap map illustrating common and distinctive brain regions for Val/Val (blue) and Val/Met (yellow) genotype patients. Overlap between Val/Val and Val/Met genotype patients is illustrated in green. In each axial slice, the right hemisphere is on the reader's left.

CT data were acquired during the Phase 3 testing period. Axial CT scans without contrast were acquired at Bethesda Naval Hospital on a GE Medical Systems Light Speed Plus CT scanner in helical mode (150 slices per subject, field of view covering head only). Images were reconstructed with an in-plane voxel size of 0.4×0.4 mm, overlapping slice thickness of 2.5 mm, and a 1 mm slice interval. Lesion location and volume were determined from CT images using the Analysis of Brain Lesion software [36], [37] contained in MEDx v3.44 (Medical Numerics) with enhancements to support the Automated Anatomical Labeling atlas [38]. Lesion volume was calculated by manual tracing of the lesion in all relevant slices of the CT image then summing the traced areas and multiplying by slice thickness. A trained neuropsychiatrist performed the manual tracing, which was then reviewed by an observer who was blind to the results of the neuropsychological testing. As part of this process, the CT image of each subject’s brain was spatially normalized to a CT template brain image. This template was created by spatial normalization of a neurologically healthy individual’s CT brain scan to MNI space using the Automated Image Registration program [39]. Lesion overlap maps for patients with the Val/Val or Val/Met genotypes are illustrated in Figures 1 and 2, respectively. Demographic and background data for the Val/Val and Val/Met patient groups are reported in Table S1 (see also [29]–[32]). No effects on test performance were observed in these patient groups on the basis of demographic variables (e.g., age, sex, ethnicity, years of education, and lesion size; see also [28]). A direct comparison of brain regions damaged in the Val/Val versus Val/Met genotypes is illustrated in Figure 3. The profile of brain damage among these patient groups primarily reflects common PFC subregions (highlighted in green) and entails the majority of damaged voxels (Val/Val = 62.02% shared with Val/Met; Val/Met = 76.33% shared with Val/Val). An additional analysis performed on a subset of the Val/Val patients whose lesion maps had maximal overlap with the full Val/Met patient group (84.94%) is reported in Table S3 and Figs. S1–S3, and replicates the findings of this study.

### Neuropsychological Tests

We administered the Wechsler Adult Intelligence Scale, 3rd Edition (WAIS; [40]) to investigate the neural substrates of key competencies for general intelligence.

### Wechsler Adult Intelligence Scale, 3^rd^ Edition

The WAIS-III comprises 14 subtests grouped in four first-order factors: verbal comprehension, perceptual organization, working memory, and processing speed. In addition, a higher-order factor representing general cognitive ability (*g*) is obtained from the variance shared by the first-order factors. Table S2 provides a brief description of each subtest (for further details concerning their standardization, reliability, and validity, see [40]).

### Confirmatory Factor Analysis

**Figure 4:**
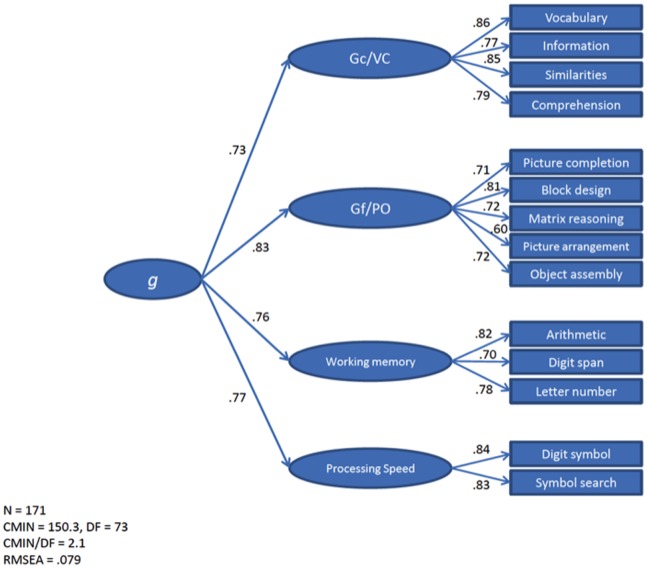
Summary of structural equation modeling results (*n* = 171).

The following measurement model was tested (Fig. 4): (1) verbal comprehension/crystalized intelligence (VC) was assessed by vocabulary, information, similarities, and comprehension subtests; (2) perceptual organization/fluid intelligence (PO) was as assessed by matrix reasoning, block design, object assembly, picture arrangement, and picture completion subtests; (3) working memory (WM) was measured by arithmetic, digit span, and letter-number sequencing subtests; (4) processing speed (PS) was measured by digit symbol coding and symbol search subtests, (5) the higher-order factor representing general intelligence (*g*) predicts the above first-order factors.

This measurement model was tested for the 171 patients using the AMOS program [41]. Fit indexes were reasonable: χ^2^ = 150.3, degrees of freedom (DF) = 73, χ^2^/DF = 2.1, RMSEA = 0.079, CFI = 0.94. All the coefficients in the model were statistically significant (*p*<0.01). Perceptual organization/fluid intelligence was the first-order factor best predicted by the higher-order factor (0.83). Nevertheless, the remaining first-order factors were also largely predicted by *g* (regression weights from 0.73 to 0.77).

Latent scores were obtained from this measurement model using the AMOS imputation algorithm. These latent scores estimate the true (error free) ability levels measured by the WAIS-III. Therefore, specific variance associated with each subtests is removed. The resulting five scores (general intelligence, fluid intelligence, crystallized intelligence, working memory, and processing speed) were submitted to voxel-based lesion-symptom mapping analysis.

## Results

Firstly, we computed scores on general cognitive ability as obtained from the Armed Forces Qualitication Test (AFQT) before TBI for Val/Val (*n* = 97) and Val/Met (*n* = 59) genotypes. A one-way ANOVA was computed and the average difference was not statistically significant (*F* = 0.364, *p* = 0.43, effect size = 0.13). Therefore, there were no average differences in general intelligence between Val/Val and Val/Met genotypes before TBI.

**Figure 5:**
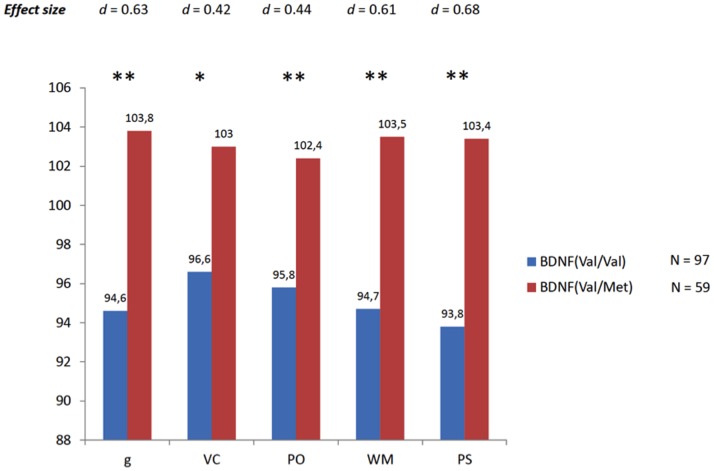
Wechsler Adult Intelligence Scale latent scores. g = general intelligence, VC = verbal comprehension, PO = perceptual organization, WM = working memory, PS = processing speed, * = *p*<0.05, ** = *p*<0.01.

Secondly, these two groups of participants were systematically compared in the five latent scores described above. Remember that WAIS-III scores were obtained after TBI. Again, a one-way ANOVA was computed. Now a substantial average difference is observed between Val/Val and Val/Met genotypes across the five latent scores (*F* = 14.9, 6.4, 7.2, 14, and 16.9 for *g*, VC, PO, WM, and PS respectively). As Figure 5 illustrates, this average difference ranges from 6 to 8 IQ points (around half a standard deviation using the standard scale with a mean of 100 and a standard deviation of 15). Values for the effect size (d) corresponding to these average differences were: 0.68 for *g*, 0.42 for VC, 0.44 for PO, 0.61 for WM, and 0.68 for PS. Therefore, while there was not a difference in general cognitive ability between both genotypes before TBI, a substantial and statistically significant average difference was noted after TBI. Val/Met genotypes preserved their cognitive ability, whereas Val/Val genotypes showed scores notably decreased.

## Discussion

The present study provides compelling evidence for a relationship between variations in the Val66Met BDNF polymorphism and preservation of general intelligence after penetrating TBI. In previous studies, the Met allele had been found to be associated with relatively impaired cognitive functions in healthy individuals [11], [13], stroke patients [42], psychiatric populations [43], [44], and in patients recovering from mild TBI [45]. However, in this study, we demonstrated that the Met allele is protective for general intelligence in patients with PFC damage after TBI. Met carriers showed preservation of general intelligence compared with Val carriers, across all facets of intelligence, including verbal comprehension, perceptual organization, working memory, and processing speed.

We investigated the relative contribution of the Met allele to the preservation of general intelligence in the presence of other factors such as age, pre-injury cognitive performance, and volume brain loss. Pre-injury cognitive performance on the Armed Forces Qualification Task (AFQT) was not different for Met or Val carriers. However, correlations between AFQT scores (before TBI) and WAIS-III scores were generally lower for Val carriers, except for verbal comprehension. This finding indicates that AFQT scores are better predictors of intelligence after TBI for the Met carries than for the Val carriers. The correlation values (AFQT and WAIS-III factors) were (Met/Val carriers): g (0.76/0.57), verbal comprehension (0.65/0.64), perceptual organization (0.77/0.64), working memory (0.70/0.56), and processing speed (0.61/0.42). Moreover, the profile of brain damage for the Met and Val carriers was comparable and did not significantly differ in terms of total percent volume loss (see Figs. 1–3).

The present study motivates new approaches to understanding the molecular mechanisms underlying the association of the Val66Met BDNF gene and preservation of general intelligence after TBI. Prior research indicates that TBI is associated with an early up regulation of BDNF in both animal models of experimental brain injury [46] and in individuals with severe TBI [47]. BDNF is synthesized in the brain as a propolypeptide, which can be processed either intracellularly or extracellularly by intracellular cleavage to mature BDNF followed by secretion, secretion followed by extracellular cleavage to mature BDNF, or secretion without subsequent cleavage [48]. Because proneurotrophins are important for proper folding, dimerization, and targeting of the mature neurotrophins, substituting Met for Val in proBDNF results in defective intracellular protein trafficking, packaging, and regulated secretion [9], [11].

ProBDNF and mature BDNF have two distinct receptors and signaling cascades resulting in opposing effects on the nervous system [48]. Whereas mature BDNF binds with high affinity to the TrK B receptor tyrosine kinases (TrkB) and insures cell survival [49], proBDNF binds with high affinity to the multifunctional p75 neurotrophin receptor (p75NTR) and triggers apoptosis [50], [51]. Importantly, proneurotrophins are upregulated in pathological conditions such as brain injury [48]. Particularly, proBDNF is secreted by neurons and glial cells [9], [51], when cell death prevails after brain trauma [52], [53]. After injury, p75NTR and many binding partners are dynamically regulated and produce unique, multimeric receptor complexes. One such binding partner is sortilin, which specifically binds the prodomain of BDNF and serves as a coreceptor with p75NTR in mediating cell death [54]. For example, when the interaction between proBDNF and sortilin/p75NTR was blocked by sortilin (a protein that is a member of the recently discovered family of Vps10p-domain receptors) antagonists, the apoptotic actions of proBDNF on cultured sympathetic neurons were abolished [51]. Moreover, lesioned corticospinal neurons with lower sortilin expression were more likely to survive the injury [55]. By extension, the reduced secretion of proBDNF Met because of impaired intracellular trafficking represents a plausible molecular model for protecting individuals with the Met allele, especially in situations when a substantial subpopulation of neurons is undergoing cell death as may occur in TBI (see also [9]). Future molecular and cellular studies on the regulation of proBDNF secretion after neuronal injury will be required to verify this proposed molecular mechanism.

Other recent research supports our observation for cognitive preservation by carriers of the Met allele. A recent study in healthy individuals revealed a positive effect of the Met allele for a cognitive control function, namely response inhibition [17]. Furthermore, the presence of the Met allele was associated with reduced cognitive decline in patients with multiple sclerosis [56] or systemic lupus erythematosus [57]. In addition, accumulating evidence in human lesion patients on the Val66Met BDNF polymorphism and cognitive function indicates that the Met allele exerts a protective effect for executive function [1] and cognitive performance following TBI [28]. Finally, recent meta-analyses of population-based case control studies on the Val66Met BDNF polymorphism and mental disorders revealed that the Met allele exerts a protective effect for substance-related disorders [26] and results in decreased neuroticism as a vulnerability trait for anxiety [27]. Thus, the weight of evidence suggests that the functional effect of the Met allele may vary between cognitive functions and brain regions under normal and pathological conditions. Recent findings further indicate that the Val66Met BDNF polymorphism does not capture all of the functionally important genetic variation in cognitive performance following TBI and emphasize the importance of investigating additional BDNF SNPs [45].

There are a number of limitations of the current study. First, in any investigation attempting to link a gene with a discrete change in cognitive function or pathology, it is often unclear how different genotypes lead to altered phenotypes. Instead of an identified genetic variant having a direct effect on executive function, it is plausible that the genetic variation mediates its effect(s) through a downstream functional change or through the regulation of some other gene. Thus, future genotyping studies are necessary to explore whether the preservation process may be mediated by other candidate genes such as those from the neurotrophic factor and TrK receptor families (see also [45]). Second, given that our performance measures were determined 30 years after TBI in participants generally in their late 50s, a number of long-term changes in BDNF expression, directly, or indirectly, could potentially impact executive function. For example, neuroprotective gene expression is altered in the elderly: TrkB mRNA levels are reduced markedly in all portions of cortex [58]. In addition, although changes in brain levels of apoptotic genes such as p75NTR remain unchanged through adulthood, sortilin levels increase with age, suggesting a mechanism that could shift the balance to neurodegeneration with increasing age [59]. In the future, longitudinal studies starting shortly after a TBI are needed to explore the long-term molecular and cellular basis of the Val66Met BDNF polymorphisms on potential recovery of executive function and other cognitive domains. Finally, although the Val/Val and Val/Met genotype groups in the present study entail a similar profile of brain damage (Fig. 3; highlighted in green) and the findings replicate when a subset of patients with maximal lesion overlap in the Val/Val and Val/Met genotype groups are compared (Table S3 and Fig. S1–S3), it remains possible that subtle differences in the location of cortical damage across these patient groups contribute to the observed differences in general intelligence following TBI (Fig. 3 and Fig. S2; regions highlighted in blue and yellow, respectively).

In conclusion, our findings provide novel evidence for a relationship between the Met allele and the preservation of general intelligence after penetrating TBI, supporting a protective effect for specific competencies of psychometric *g*, including verbal comprehension, perceptual organization, working memory, and processing speed. For current clinical application, earlier triage and extended cognitive rehabilitation is recommended for carriers of the Val/Val allele to facilitate the best possible long-term social and vocational outcomes for patients with PFC damage after TBI.

## Supporting Information

Figure S1 **Lesion mapping results for Val/Val focal genotype patients (** ***n*** **= 59).** In each axial slice, the right hemisphere is on the reader’s left.
(TIFF)

Figure S2 **Lesion overlap map illustrating common and distinctive brain regions for Val/Val Focal (blue) and Val/Met (yellow) genotype patients.** Overlap between Val/Val Focal and Val/Met genotype patients is illustrated in green. In each axial slice, the right hemisphere is on the reader's left.
(TIFF)

Figure S3 **Mean performance for Wechsler Adult Intelligence Scale latent scores for a subset of 59 Val/Val patients that share 84.98% of its voxels with the full Val/Met patient group.** g = general intelligence, VC = verbal comprehension, PO = perceptual organization, WM = working memory, PS = processing speed.
(TIFF)

Table S1 **Demographic and background data.** Note: “Age” refers to age at the time of Phase 3 evaluation. “Ethnicity” refers to the percentage of Caucasian veterans. “Sex” refers to the percentage of male veterans. “Years of education” refers to the total number of years of education the veterans completed. “Total percent volume loss” refers to the total percent volume loss due to brain damage in cm^3^.
(TIFF)

Table S2 **Description of intelligence measures of the WAIS.**
(TIFF)

Table S3 **Mean performance with effect sizes for Wechsler Adult Intelligence Scale latent scores on a subset of 59 Val/Val patients that share 84.98% of its voxels with the full Val/Met patient group.** g = general intelligence, VC = verbal comprehension, PO = perceptual organization, WM = working memory, PS = processing speed.
(TIFF)
