# Supplementary figures and images for: Preservation of General Intelligence following Traumatic Brain Injury: Contributions of the Met66 Brain-Derived Neurotrophic Factor
Source: PLoS One. 2014 Feb 26;9(2):e88733. doi: 10.1371/journal.pone.0088733 (PMC3935849; doi:10.1371/journal.pone.0088733)

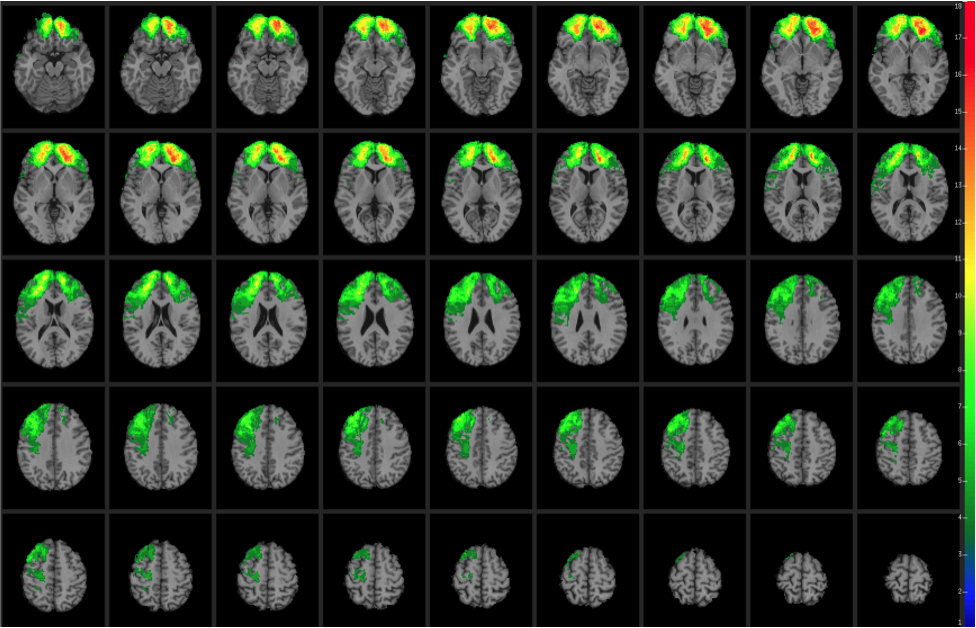

Supplement: Figure S1 — Lesion mapping results for Val/Val focal genotype patients ( n = 59). In each axial slice, the right hemisphere is on the reader’s left. (TIFF) [file pone.0088733.s001.tif]

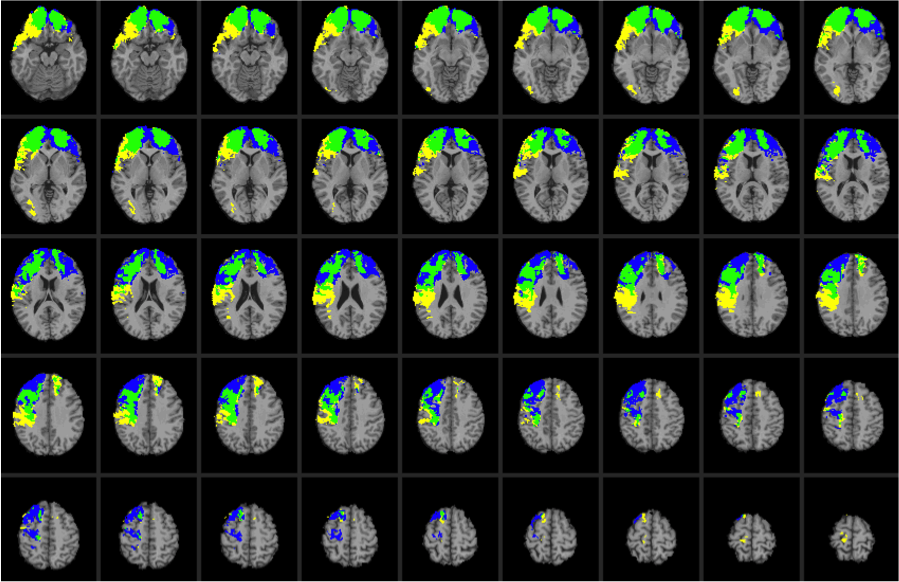

Supplement: Figure S2 — Lesion overlap map illustrating common and distinctive brain regions for Val/Val Focal (blue) and Val/Met (yellow) genotype patients. Overlap between Val/Val Focal and Val/Met genotype patients is illustrated in green. In each axial slice, the right hemisphere is on the reader's left. (TIFF) [file pone.0088733.s002.tif]

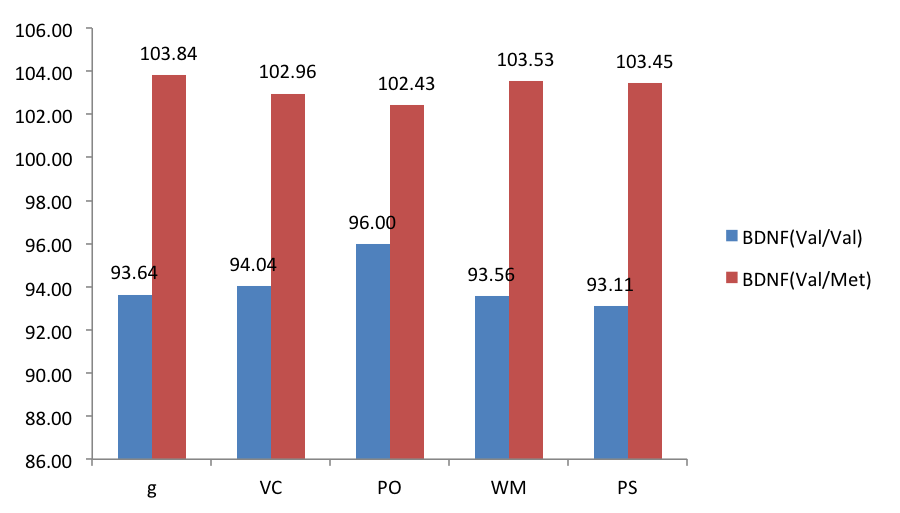

Supplement: Figure S3 — Mean performance for Wechsler Adult Intelligence Scale latent scores for a subset of 59 Val/Val patients that share 84.98% of its voxels with the full Val/Met patient group. g = general intelligence, VC = verbal comprehension, PO = perceptual organization, WM = working memory, PS = processing speed. (TIFF) [file pone.0088733.s003.tif]

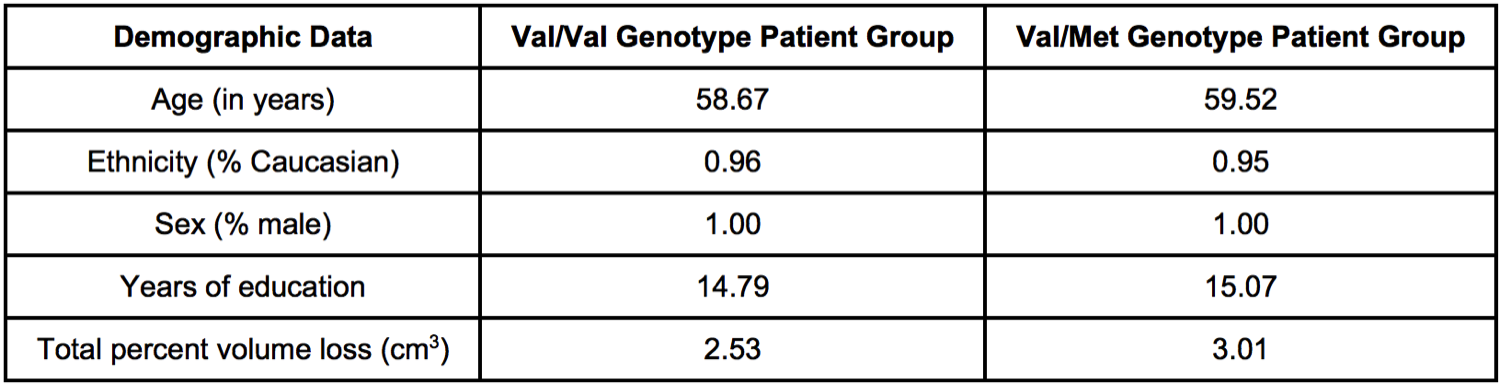

Supplement: Table S1 — Demographic and background data. Note: “Age” refers to age at the time of Phase 3 evaluation. “Ethnicity” refers to the percentage of Caucasian veterans. “Sex” refers to the percentage of male veterans. “Years of education” refers to the total number of years of education the veterans completed. “Total percent volume loss” refers to the total percent volume loss due to brain damage in cm3. (TIFF) [file pone.0088733.s004.tif]

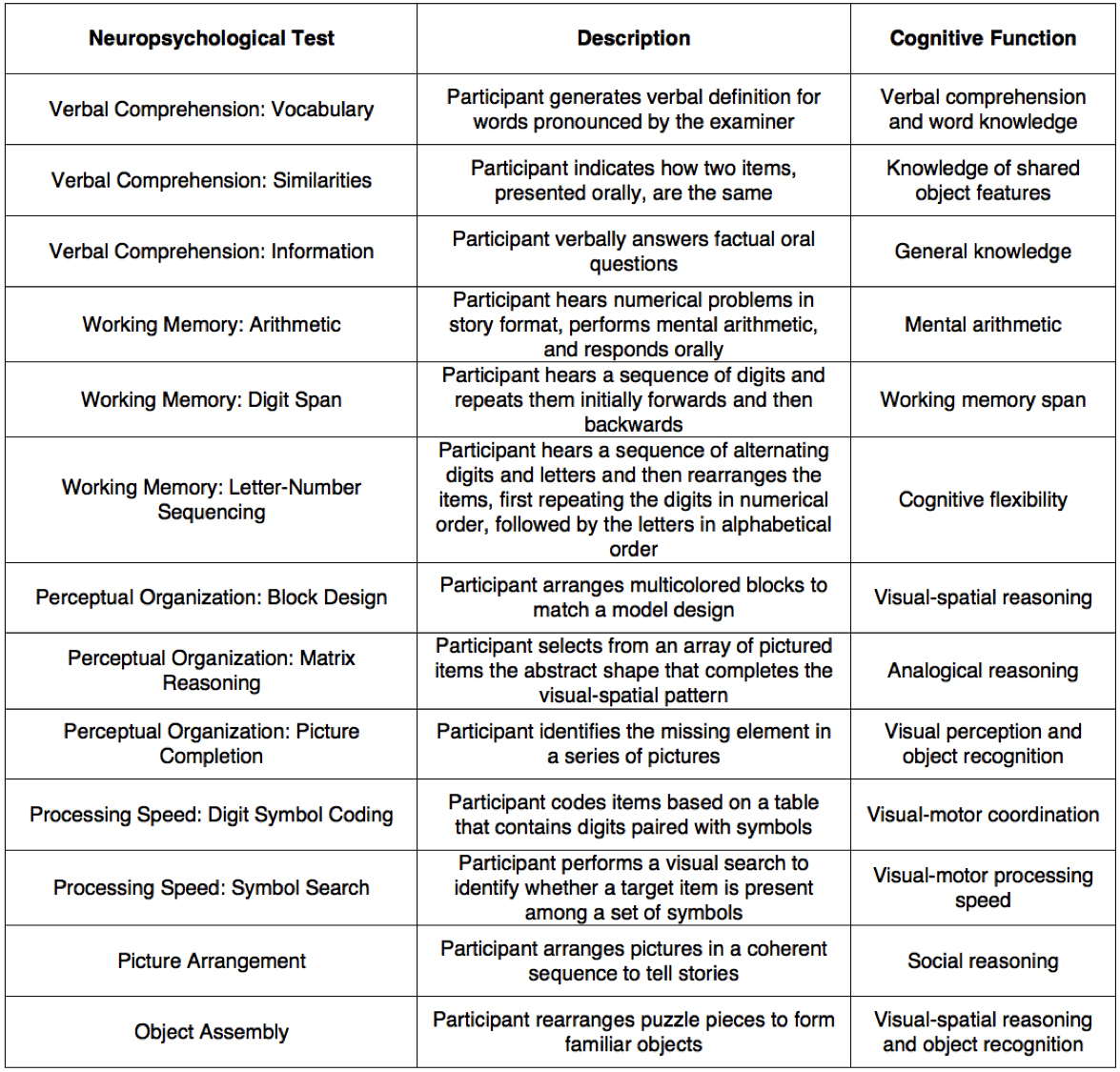

Supplement: Table S2 — Description of intelligence measures of the WAIS. (TIFF) [file pone.0088733.s005.tif]

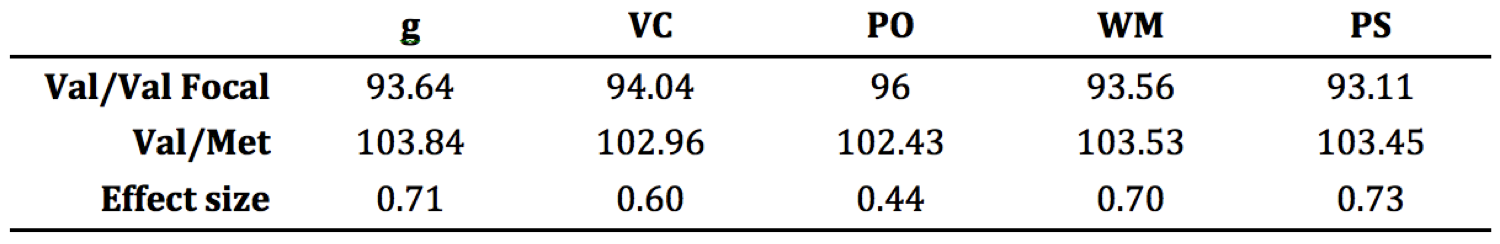

Supplement: Table S3 — Mean performance with effect sizes for Wechsler Adult Intelligence Scale latent scores on a subset of 59 Val/Val patients that share 84.98% of its voxels with the full Val/Met patient group. g = general intelligence, VC = verbal comprehension, PO = perceptual organization, WM = working memory, PS = processing speed. (TIFF) [file pone.0088733.s006.tif]
